# Fine-Scale Mapping of the Nasonia Genome to Chromosomes Using a High-Density Genotyping Microarray

**DOI:** 10.1534/g3.112.004739

**Published:** 2013-02-01

**Authors:** Christopher A. Desjardins, Jürgen Gadau, Jacqueline A. Lopez, Oliver Niehuis, Amanda R. Avery, David W. Loehlin, Stephen Richards, John K. Colbourne, John H. Werren

**Affiliations:** * Department of Biology, University of Rochester, Rochester, New York 14627; † Broad Institute, Cambridge, Massachusetts 02141; ‡ School of Life Sciences, Arizona State University, Tempe, Arizona 85287; § The Center for Genomics and Bioinformatics, Indiana University, Bloomington, Indiana 47405; ** Center for Molecular Biodiversity Research, Zoological Research Museum Alexander Koenig, 53113 Bonn, Germany; †† Human Genome Sequencing Center, Baylor College of Medicine, Houston, Texas 77030

## Abstract

Nasonia, a genus of four closely related parasitoid insect species, is a model system for genetic research. Their haplodiploid genetics (haploid males and diploid females) and interfertile species are advantageous for the genetic analysis of complex traits and the genetic basis of species differences. A fine-scale genomic map is an important tool for advancing genetic studies in this system. We developed and used a hybrid genotyping microarray to generate a high-resolution genetic map that covers 79% of the sequenced genome of *Nasonia vitripennis*. The microarray is based on differential hybridization of species-specific oligos between *N. vitripennis* and *Nasonia giraulti* at more than 20,000 markers spanning the Nasonia genome. The map places 729 scaffolds onto the five linkage groups of Nasonia, including locating many smaller scaffolds that would be difficult to map by other means. The microarray was used to characterize 26 segmental introgression lines containing chromosomal regions from one species in the genetic background of another. These segmental introgression lines have been used for rapid screening and mapping of quantitative trait loci involved in species differences. Finally, the microarray is extended to bulk-segregant analysis and genotyping of other Nasonia species combinations. These resources should further expand the usefulness of Nasonia for studies of the genetic basis and architecture of complex traits and speciation.

The parasitic wasp genus Nasonia (Insecta: Hymenoptera) is a model system in genetics, particularly evolutionary genetics, the genetics of complex traits, developmental genetics, and host−parasite interactions (Beukeboom and Desplan 2003; Werren and Loehlin 2009). Nasonia consists of four closely related species, *Nasonia vitripennis*, *Nasonia giraulti*, *Nasonia longicornis*, and the recently discovered *Nasonia oneida*, which are all cross-fertile once cured of their endosymbiotic Wolbachia (Breeuwer and Werren 1995; Raychoudhury *et al.* 2010). The presence of interfertile species is advantageous for evolutionary genetic research because it allows movement of genetic regions between species for the identification, mapping, and cloning of quantitative trait loci (QTL) involved in species differences. Several additional features make Nasonia an excellent genetic model, including short (2-wk) generation time, ease of laboratory rearing, systemic RNA interference, and haplodiploid sex determination (females are diploid, whereas males are haploid and derived from unfertilized eggs, facilitating mutation screening) (Lynch and Desplan 2006; Werren and Loehlin 2009; Werren *et al.* 2009). The *N. vitripennis−N. giraulti* species pair has been used widely to identify QTL involved in a diverse array of phenotypes, including wing size (Gadau *et al.* 2002; Loehlin *et al.* 2010a,b; Loehlin and Werren 2012), cuticular hydrocarbons (Niehuis *et al.* 2011), hybrid incompatibilities (Breeuwer and Werren 1995; Gadau *et al.* 1999; Gibson *et al.* 2010; Koevoets *et al.* 2012; Niehuis *et al.* 2008; Werren *et al.* 2010), and host preference (Desjardins *et al.* 2010).

The recent sequencing of the genomes of three Nasonia species (Werren *et al.* 2010) provides a key resource for advancing this new model system. Using the genome sequence data in conjunction with haploid males from hybrid crosses between *N. vitripennis* and *N. giraulti*, a genetic map of the Nasonia genome was generated (Niehuis *et al.* 2010; Werren *et al.* 2010). This approach was facilitated by the high level of single-nucleotide polymorphisms (SNPs) between species (average coding sequence difference between *N. vitripennis* and *N. giraulti* is 1%). The mapping population consisted of haploid male embryos from F_1_ hybrid females. The advantage of hybrid haploid males is that for any locus, a hybrid male carries only the allele for one of the parental species. With the use of this strategy, 265 scaffolds (covering 64% of the assembled genome) were mapped onto the five chromosomes of Nasonia (Niehuis *et al.* 2010). The combination of a genome sequence and genetic map has allowed investigators studying Nasonia to do forward genetics efficiently, *i.e.*, quickly proceed from detection of QTL to positional cloning of QTL and identification of the genetic architecture that underlie phenotypes of interest (Loehlin *et al.* 2010b; Loehlin and Werren 2012).

To further advance Nasonia as a genetic system, a high-resolution genetic map is needed that places more scaffolds onto the linkage map and has a finer scale of resolution. In addition, the availability of a cost-effective tool for high-throughput genotyping and bulk-segregant analysis could help advance studies geared toward investigating the genetic basis of complex adaptive phenotypes, genetic incompatibilities, and species differences. Here, we developed a comparative hybridization genotyping (CGH) microarray that uses clusters of SNPs and insertion-deletions (indels) in a high-density genotyping microarray to differentiate *N. vitripennis* from *N. giraulti* sequence at a large number of markers spanning the Nasonia genome.

Additional resources being developed for Nasonia include a set of segmental introgression lines (SILs), which contain specific genomic regions from one species (typically *N. giraulti*) in the genetic background of another (typically *N. vitripennis*) (Werren and Loehlin 2009). These are produced by performing an interspecific cross followed by repeated backcrossing of hybrid females to males of one species. To “hold onto” the introgressed genetic region during the backcrossing process, either visible mutant markers, phenotypic species-differences (such as wing size), or molecular markers are used. Eventually (usually greater than eight generations depending on the size of the introgressed region) the introgression lines are made homozygous for the “foreign” target region within the other species’ genetic background. SILs already have been used for efficient mapping of genes affecting phenotypic differences between Nasonia species (Loehlin *et al.* 2010b; Loehlin and Werren 2012). To further develop Nasonia SIL resources for QTL mapping, here we used the microarray to genotype 26 SILs.

We also tested the applicability of the microarray to bulk segregant analysis, another useful tool for efficiently identifying genomic regions associated with phenotypes of interest (Michelmore *et al.* 1991). In bulk segregant analysis, a population is divided into two subsamples based on phenotype, and then allele proportions are estimated for each subsample. It is an alternative to genotyping a large population at the level of individuals, and is an economical way to quickly and efficiently identify genomic regions associated with traits of interest. For example, this approach was used in combination with genotyping to identify regions associated with nuclear-cytoplasmic incompatibility in hybrids of *N. vitripennis* and *N. giraulti* (Werren *et al.* 2010). Here we broadly applied the methodology to mapping phenotypes in Nasonia by using the CGH microarray.

The CGH array will be of even greater utility if it can be applied to hybrid crosses involving other Nasonia species, such as *N. longicornis* and *N. oneida*, as a number of research groups are currently investigating these species (L. Beukeboom, personal communication; Koevoets 2012). Although the array was not designed for this purpose, the close phylogenetic relationship of both *N. longicornis* and *N. oneida* to *N. giraulti* suggest that their DNA would be more likely to hybridize to the *N. giraulti* oligo than the *N. vitripennis* oligo (Raychoudhury *et al.* 2010). We therefore tested how many markers on the microarray can accurately distinguish between *N. vitripennis−N. longicornis* and *N. vitripennis−N. oneida* sequences.

## Methods and Materials

### Strains used

Crosses to generate the F_2_ mapping population were between the *N. vitripennis* (ASymCX) and *N. giraulti* (RV2Xu) strains used in the genome sequencing project (Werren *et al.* 2010). SILs were generated by sequential backcrossing for variable numbers of generations (usually more than eight) and then typically made homozygous. Some lines were subsequently subjected to additional backcrosses to further reduce the size of the introgressed segment. Some SILs have been described in previous studies, whereas others are first reported here and were generated in the Werren laboratory (University of Rochester) as a resource for QTL mapping and cloning (Table 1). Typical nomenclature refers to the marker (visible or molecular) used in the introgression with subscript for the species of the allele, followed by capital letters for the genetic background it was introgressed into (*e.g.*, rdh5_g_V for the *rdh5* eye color marker wild-type allele from *N. giraulti* in a *N. vitripennis* genetic background), with V representing *N. vitripennis* and G representing *N. giraulti*. The introgression lines will contain the marker used plus introgressed flanking DNA and in some cases unlinked regions that also were retained during the introgression.

**Table 1:** Descriptions of SILs

Figure Code	Introgression Name	Introgressed Locus	Introgressed/ Background Species	Starting Cluster	Starting Marker	Ending Cluster	End Marker	Introgression Size, bp	Locus Citation
a	SIL02_ant_gV	antennapedia mutant	g/V	1.004	SCAFFOLD_16_695042_695121	1.026	SCAFFOLD_16_3632344_3632423	3006600	(Werren and Perrot-Minnot 1999)
b	SIL03_rdh5_gV	*rdh5* eye color	g/V	1.061	SCAFFOLD_33_665868_665947	1.063	SCAFFOLD_1_631906_631985	1928226.5	(Saul 1990)
c	SIL04_b3u_gV	*wdw* wing QTL	g/V	1.096	SCAFFOLD_1_4594963_4595042	1.107	SCAFFOLD_7_7419_7498	1651579	(Loehlin and Werren 2012)
d	SIL05_d1_gV	*wdw* wing QTL	g/V	1.091	SCAFFOLD_1_3613637_3613716	1.106	SCAFFOLD_7_24252_24331	2610217	(Loehlin and Werren 2012)
e	SIL06_hsb3u_gV	*wdw* wing QTL	g/V	1.096	SCAFFOLD_1_4594963_4595042	1.107	SCAFFOLD_7_7419_7498	1651579	(Loehlin and Werren 2012)
f	SIL07_wdw_vG	*wdw* wing QTL	v/G	1.072	SCAFFOLD_1_1849534_1849613	1.102	SCAFFOLD_1_5660392_5660471	3823298.5	(Loehlin and Werren 2012)
g	SIL08_st318_gV	*st-318* eye color	g/V	3.043	SCAFFOLD_89_601954_602033	3.077	SCAFFOLD_17_1447204_1447283	24316119.5	(Saul 1990)
h	SIL09_stmm_gV	*st-318*,mm head shape	g/V	3.056	SCAFFOLD_22_1563155_1563234	3.087	SCAFFOLD_17_2553865_2553944	3239862.5	(Saul 1990)
i	SIL10_bk424_gV	*bk424* eye color	g/V	4.000	SCAFFOLD_4_5206596_5206675	4.007	SCAFFOLD_4_3692382_3692461	1585134.5	(Saul 1990)
j	SIL11_mrp901_gV	mitochondrial riboprotein 901 molecular marker	g/V	4.016	SCAFFOLD_4_3019664_3019743	4.028	SCAFFOLD_4_1505972_1506051	1519922.5	J. H. Werren, unpublished data
k	SIL12_wbr055_gV	*ws1* wing QTL	g/V	4.036	SCAFFOLD_4_325612_325691	4.051	SCAFFOLD_23_136201_136280	2631402	(Loehlin *et al.* 2010b)
l	SIL13_w1.1_gV	*ws1* wing QTL	g/V	4.037	SCAFFOLD_4_290478_290557	4.051	SCAFFOLD_29_160960_161039	2898380.5	(Loehlin *et al.* 2010b)
m	SIL14_bkbw_gV	*ws1* wing QTL	g/V	4.047	SCAFFOLD_23_1874450_1874529	4.051	SCAFFOLD_29_160960_161039	2069456	(Desjardins *et al.* 2010)
				4.056	SCAFFOLD_17_431985_432064	4.069	SCAFFOLD_40_1771694_1771773	21850167.5	
n	SIL15_ws1_844_gV	*ws1* wing QTL	g/V	4.047	SCAFFOLD_23_1874450_1874529	4.051	SCAFFOLD_29_160960_161039	2069456	(Loehlin *et al.* 2010b)
				4.056	SCAFFOLD_17_431985_432064	4.062	SCAFFOLD_66_570309_570388	5341440.5	
o	SIL16_ws1_vG	*ws1* wing QTL	g/V	4.056	SCAFFOLD_23_1661132_1661211	4.055	SCAFFOLD_35_1537323_1537402	8091567	(Loehlin *et al.* 2010b)
p	SIL17_ws1_lV	*ws1* wing QTL	g/V	4.051	SCAFFOLD_23_562542_562621	4.063	SCAFFOLD_66_570309_570388	12358488	(Loehlin *et al.* 2010b)
q	SIL18_s6k_gV	s6 kinase molecular marker	g/V	4.057	SCAFFOLD_17_317904_317983	4.061	SCAFFOLD_51_686510_686589	4328932	J. H. Werren, unpublished data
r	SIL19_sww845_gV	shorter wider wing QTL	g/V	4.064	SCAFFOLD_66_364114_364193	4.068	SCAFFOLD_40_1838710_1838789	16310977	J. H. Werren, unpublished data
s	SIL20_sww_gV	shorter wider wing QTL	g/V	4.065	SCAFFOLD_43_100411_100490	4.071	SCAFFOLD_40_1699997_1700076	15969580.5	J. H. Werren, unpublished data
t	SIL21_ws2_gV	*ws2* wing QTL	g/V	4.087	SCAFFOLD_9_3481449_3481528	4.103	SCAFFOLD_9_42054_42133	3498562	J. H. Werren, unpublished data
u	SIL22_nadhR	NADH-ubiquitin molecular marker	g/V	5.000	SCAFFOLD_14_23509_23588	5.001	SCAFFOLD_14_627130_627209	637381.5	J. H. Werren, unpublished data
v	SIL23_atpd4_gV	Atpase-6 molecular marker	g/V	5.003	SCAFFOLD_14_1325752_1325831	5.015	SCAFFOLD_38_1386740_1386819	2548446.5	J. H. Werren, unpublished data
w	SIL24_PePu_GDgV	peach eye, purple body, *grounded* (tiny wing)	g/V	5.008	SCAFFOLD_14_2690831_2690910	5.017	SCAFFOLD_7_3061899_3061978	3542691.5	(Saul 1990; Werren *et al.* 2010)
x	SIL25_pegpugVgd	peach eye, purple body	g/V	5.028	SCAFFOLD_7_1286368_1286447	5.044	SCAFFOLD_2085_9762_9841	12894737.5	(Saul 1990; Werren *et al.* 2010)
y	SIL26_stdr109_gV	*st-DR* eye color	g/V	5.041	SCAFFOLD_1_9390701_9390780	5.055	SCAFFOLD_10_2982477_2982556	12400393	(Saul 1990)
z	SIL27_stdr_gV	*st-DR* eye color	g/V	5.041	SCAFFOLD_1_9390701_9390780	5.071	SCAFFOLD_2_4551481_4551560	16697318	(Saul 1990)

SILs, segmental introgression lines. Shown is the reference code for Figure 3 (a graphical depiction of SIL genotypes), name of the introgression, locus used in the introgression, introgressed and background species (V for *N. vitripennis* or G for *N. giraulti)*, location of the introgression, introgression size, and relevant citation for the loci used in the introgression. Note that the introgression names show the species genotype of the introgressed region as a small letter (v, g or l for *N. longicornis*) and the background species as a large letter (V or G).

### Microarray design

To identify polymorphisms (SNPs and small indels of 1–50 bp), we used *N. giraulti* genomic sequence (1X Sanger) aligned to the *N. vitripennis* genome sequence (Werren *et al.* 2010). Based on these alignments, we identified 80-bp segments with clusters of fixed differences between the two genomes. Sequence segments were filtered to identify those with a SNP at the 3′ end of the sequence, at least five polymorphisms in the 3′ most 20 bp, and at most 15 polymorphisms in the entire 80 bp length. A preponderance of SNPs at the 3′ region was preferred because the oligos are attached to the array substrate at the 5′ end, and solution-end polymorphisms have been shown to be more important to differential hybridization than polymorphisms near the substrate end (Hughes *et al.* 2001). Individual bases within a single insertion or deletion were each counted as polymorphisms (*e.g.*, a three-base insertion was counted as three polymorphisms). To increase confidence in polymorphism calls, at least 2× coverage of the *N. giraulti* sequence was required along the entire 80-bp segment. All SNPs were required to have PHRED quality values ≥50, and no intraspecific polymorphism was allowed. Finally, the 70 bp downstream of the selected sequence was allowed to contain a maximum of four SNPs to ensure proper alignment of the entire region. Downstream sequences with no SNPs were retained as potential control sequences for the array. In addition to polymorphism clusters, we identified moderately sized indels (30–50 bp) in the alignments and selected 80 bp of total sequence for each species including the insertion when present and flanking sequence centered on the middle of the indel.

From the resulting sequence files, we selected 70-bp subsequences to be potential oligos by using Oligopicker 2.3.2 (Wang and Seed 2003). Oligo sequences were then trimmed from the 5′ end so that they would require no more than 180 synthesis cycles according to the Roche NimbleGen protocol. We then removed all oligos with multiple matches to the genome or any number of matches to Nasonia mitochondrial DNA as determined by WU-BLAST (Lopez *et al.* 2003) (e = 1 × 10^−5^, hspsepsmax = 500). From the remaining set, only pairs with both an *N. vitripennis* and *N. giraulti* oligo were retained.

The microarray was manufactured by Roche NimbleGen’s custom design service and consists of all probes on each of four subarrays, which can altogether genotype up to eight samples simultaneously using two-color labeling and hybridization. The same microarray was reused three times by stripping the labeled targets after each hybridization and scanning. Data for custom assembly of the CGH microarray and SNP calling of oligos from the array are available at request from the authors.

### DNA preparation

For mapping, F_2_ hybrid haploid male embryos were used to minimize mapping artifacts due to postembryonic mortality of certain species-hybrid genotypes (Breeuwer and Werren 1995; Niehuis *et al.* 2008). The method to isolate and amplify DNA from embryos has been described in detail elsewhere (Niehuis *et al.* 2008, 2010). For microarray hybridization of DNA from individual adult Nasonia, including SILs, DNA was extracted using the QIAGEN Puregene Gentra protocol for single Drosophila (http://www.qiagen.com/literature). For microarray hybridization of DNA for bulk sample studies, Nasonia were pooled into a single tube and DNA was then extracted using the QIAGEN Puregene Gentra protocol for 1–30 Drosophila, with two Nasonia equaling one Drosophila. All resulting DNA from both individual and bulk samples was subsequently amplified using the GenomiPhi DNA amplification kit (Amersham Biosciences, Piscataway, NJ). DNA was quantified using a Qubit Fluorometer (Life Technologies).

### DNA labeling and hybridization

DNA was labeled and hybridized following Roche NimbleGen’s User’s Guide: CGH Analysis v.3.0 methods with minor modifications. At least 1.0 µg of dsDNA was fragmented to 1000–2000 bp in length (as determined by gel electrophoresis) by either hydrodynamic shearing (HydroShear, GeneMachines) or sonication (Sonicator 4000, Misonix). Genomic DNA with lengths primarily between 2000 and 10,000 bp based on gel electrophoresis analysis was subject to hydrodynamic shearing (which offers greater fragmentation sensitivity and control), whereas DNA with lengths >10,000 bp was fragmented by means of sonication. Fragmented DNA was labeled by random primer labeling. First, 1.0 µg of fragmented DNA was primed with 1 O.D. Cy-labeled random monomer primer (Trilink Biotechnology) at 98° for 10 min and subsequently chilled at 0°–2° for 15 min. Next, 100 U Klenow Fragment (3′ > 5′ exo-) and 10 mM dNTP mix (to final concentration of 1 mM dNTPs) was added to the primed DNA. The mixture was incubated at 37° in a thermocycler (heated-lid) for 2 hr and then stopped with 0.05 M EDTA (final concentration). Cy-labeled DNA was purified by isopropanol precipitation in the presence of sodium chloride (0.5 M NaCl final concentration). Dual-color hybridization, posthybridization washing, and scanning were performed according to manufacturer’s instructions (Roche NimbleGen’s User’s Guide: CGH Analysis v.3.0). Images were acquired using a GenePix Professional 4200A scanner with the GenePix 6.0 software. The data from these arrays were saved and exported using the software NimbleScan 2.4 (Roche NimbleGen Inc.).

### Calculation of differential hybridization and genotype calling

To calculate a normalized difference in hybridization of a DNA sample to paired species-specific oligos, a modified version of M and A values from two-color cDNA microarray analyses was used. We calculated this normalized difference in hybridization (“hybridization value”) as (intensity to *N. vitripennis* oligo minus the intensity to *N. giraulti* oligo) divided by (intensity to *N. vitripennis* oligo plus the intensity to *N. giraulti* oligo), resulting in numbers ranging from −1 to 1. Positive numbers indicate better hybridization to the *N. vitripennis* oligo than to the *N. giraulti* oligo, whereas negative numbers indicate the opposite. We then tested the array by hybridizing DNA from seven *N. vitripennis* (strain ASymCX) and seven *N. giraulti* (strain RV2Xu) single whole adult females. Both strains were used for the Nasonia genome sequencing project (Werren *et al.* 2010). We then calculated mean, median, and SD of the hybridization values for each studied marker (the genomic region represented by an oligo pair) as a quality control measure.

To genotype a marker of a test individual, we calculated the cumulative probability that the marker belonged to each genotype, *N. vitripennis* and *N. giraulti*, based on the pure species hybridization value, assuming the distribution of hybridization values for a single marker within a species is normally distributed. Markers were then called as *N. vitripennis* (V), *N. giraulti* (G), or ambiguous (U) based on three parameters (supporting information, File S1). We optimized all parameters relative to precision (the probability that a called genotype is correct) and recall (the probability that an unambiguous genotype is called for a given locus, also referred to as sensitivity), resulting in 99.5% precision at a level of 90.8% recall (File S1, Figure S1, Table S6).

In an attempt to further increase precision, we developed a smoothing algorithm based on genotypes of neighboring markers. If the two closest neighbors of a marker both have at least one opposite genotype call and have no similar genotype calls, that marker is converted to ambiguous. If a marker has only one neighbor, and the two markers are called opposite genotypes, both are considered ambiguous. If a marker has no neighbors (*e.g.*, scaffolds with only one marker), no correction can be made. Optimization of parameters using this algorithm resulted in an increased precision of 99.9% and recall of 97.7% (File S1, Figure S1, Table S6).

To determine whether the array could successfully distinguish other species pairs, DNA from four *N. longicornis* and five *N. oneida* females was individually extracted and hybridized to the array as described previously. Hybridization value statistics were then recalculated for each marker for each comparison, and precision and recall was calculated using the *N.vitripennis*−*N. longicornis* SIL (SIL17_ws1_lV; Table 1) in the same manner as the initial parameter optimization.

### Mapping analysis

To estimate recombination frequencies and generate a linkage map, we genotyped the same 110 F_2_ hybrid male embryos previously used to generate a linkage map with fewer markers (Niehuis *et al.* 2010), plus an additional 27 newly generated F_2_ hybrid male embryos. All embryos were derived from virgin F_1_ hybrid females generated by an initial hybrid cross between *N. vitripennis* (ASymCX) females and *N. giraulti* (RV2Xu) males. The two parental strains also were used for genome sequencing and should therefore provide the best sequence fit to the oligos used on the array (Werren *et al.* 2010). F_2_ hybrid male embryos were extracted from the fly host as described previously (Niehuis *et al.* 2008).

As the construction of a linkage map requires extremely accurate genotype profiles, we opted to use a set of 19,681 markers and parameters with a precision of 99.98%. To avoid an inflation of recombination estimates due to genotyping errors not correctable by smoothing, we excluded all scaffolds represented by only one marker. We genotyped each hybrid for all remaining markers, and then collapsed these loci into a set of marker clusters with unique genotype profiles. Markers that could not be unambiguously placed into a single cluster were subsequently excluded from map construction. This resulted in 498 clusters composed of 15,546 markers. Using the clusters, we generated a preliminary linkage map using the program MultiPoint (www.multiqtl.com) (Mester *et al.* 2003). Clusters used for linkage mapping showed no significant segregation distortion, and none of the genotype profiles used for creating the map was missing a genotype. The initial two-point mapping was performed with the minimum recombination frequency setting of 0.18, which resulted in five linkage groups containing all clusters. The initial marker cluster order within a linkage group was verified using a jackknifing procedure (Mester *et al.* 2003).

We further refined the linkage map by removing genotypes that required double recombinants between physically close marker clusters by using the “graphical genotypes” feature in Multipoint, as the probability of a double recombinant over a short physical distance in an individual is extremely low or impossible due to genetic interference (Berchowitz and Copenhaver 2010; Muller 1916; Sturtevant 1915). Once these genotypes were removed, Multipoint was rerun to produce an improved linkage map. In a last step to improve the marker cluster order of the linkage map, we examined the linkage map for potential mis-assembled contigs and scaffolds in the *N. vitripennis* assembly version 1.0. In cases where a potential assembly error was only supported by a single genotyped sample and by two or fewer consecutive genotypes, we corrected the linkage map to agree with the assembly. In all other cases, the inferred linkage map was considered correct, indicating that the corresponding contig or scaffold is very likely mis-assembled. Finally, we used recombination events in SILs to subdivide clusters and orient some scaffolds within those clusters.

Once the linkage map was complete, we estimated the positions of 4136 previously excluded markers on the linkage map. These included markers on scaffolds represented by a single marker, and markers with genotyping profiles that could not be unambiguously assigned to single cluster. We identified all marker clusters that had the best matches to genotype profiles of the excluded markers, allowing a maximum of three mismatches.

### Bulk segregant analysis

We tested the applicability of bulk segregant analysis using the microarray on a sample composed of individuals with known genotypes. Using two linked *N. vitripennis* mutations, *st-318* (scarlet eyes) and *mm* (“mickeymouse,” bulging eyes), we backcrossed wild-type *N. giraulti* (*st-318^+g^*,*mm^+g^*) in a *st-318*, *mm* mutant *N. vitripennis* background for eight generations. The resulting heterozygous females were set as virgins to produce haploid male offspring segregating for the mutant phenotypes. This created a population of individuals whose *N. giraulti* allele frequency across the region should be correlated with the distance from the visible markers. From these offspring, 24 recombinant males were selected from each recombinant class (*st-318^+g^*,*mm* and *st-318*,*mm^+g^*).

We separated the heads and bodies of each individual, and the bodies of each of the two recombinant classes were pooled. DNA was extracted from each pool and subsequently amplified using a GenomiPhi amplification kit (described previously). The two pools were then independently hybridized to the array and genotype frequencies of the samples were calculated based on the relative hybridization to alternate oligos. Assuming the proportion of alleles in a pooled sample of *N. vitripennis*−*N. giraulti* hybrids follows a uniform distribution between the median hybridization values of pure *N. vitripennis* and *N. giraulti* samples, the proportion of the DNA hybridizing to the allele from each respective species can be estimated for every marker. To test the accuracy of the bulk segregation results, DNA was extracted from individual heads and genotyped at four markers known to be linked to the mutations using polymerase chain reaction (PCR), as described in Werren *et al.* (2010).

## Results and Discussion

### A high-density genotyping microarray

The designed microarray includes polymorphism-cluster oligo pairs representing 19,708 markers on a total of 933 scaffolds, which cover 255 of the 295 Mb (86%) of the assembled genome. This includes 100% of scaffolds greater than 400 Kb (139/139) and 87% of scaffolds greater than 100 Kb (404/467). Of these 19,708 markers, 1696 also have a control oligo from sequence located immediately downstream of the marker. Additionally, the array includes large-indel oligo pairs representing 843 markers within those same scaffolds, for a total of 20,551 markers. Both scaffold coverage and marker density are significant improvements over the previous single-SNP-based microarray, which covered only 265 scaffolds (64%) of the genome and had 1,255 single-SNP-based probes.

The distribution of markers between coding and non-coding DNA is shown in Table 2. The majority of markers (88%) fall entirely within either non-coding DNA or introns, although 1096 markers fall entirely within exons. Nevertheless, most annotated genes in *N. vitripennis* are in close proximity to a mapped marker; of the 10,734 genes in the *N. vitripennis* RefSeq gene set in Genbank, 94% are on a scaffold represented on the microarray, and those genes are on average 7.4 kb from the nearest marker. This means that mapped markers (and associated SNPs) can be used for microdissection of QTL to regions containing one or a few genes.

**Table 2:** Distribution of oligo pairs between coding and non-coding DNA

Location	Number of Oligos
Within an exon	1096
Within an intron	7377
Crosses intron-exon boundary	982
Crosses gene-intergene boundary	214
Intergenic	10,039

**Figure 1:**
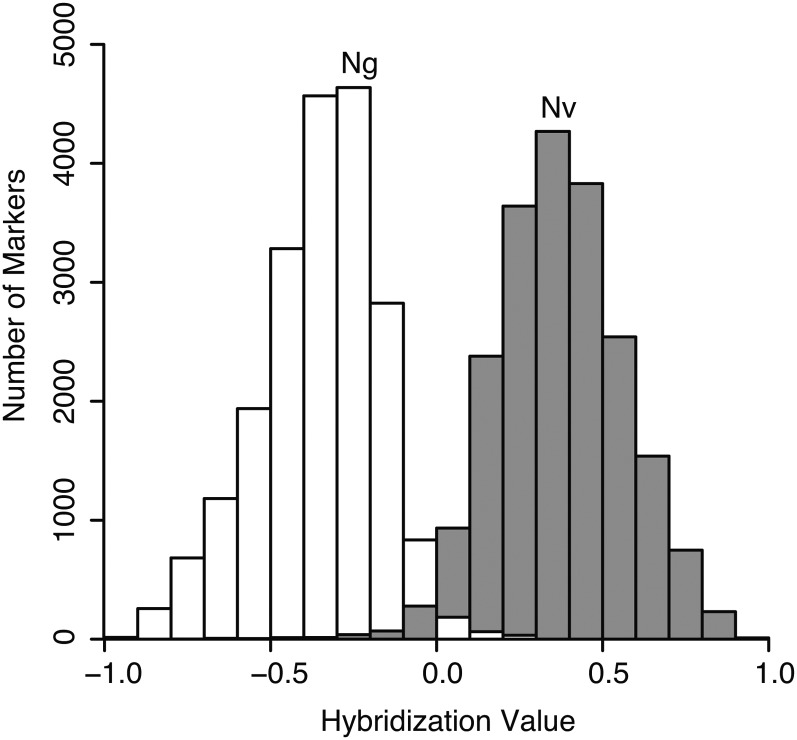
Frequency histograms of hybridization values when hybridized with *N. vitripennis* (Nv; gray) or *N. giraulti* (Ng; white) control DNA. The Y-axis shows the number of markers which produced the relevant hybridization values of the 20,551 total markers. Hybridization values > 0 indicate greater hybridization to the *N. vitripennis* oligo, whereas values < 0 indicate greater hybridization to the *N. giraulti* oligo. Only a small fraction of markers produce greater hybridization to the alternate species oligo when hybridized with control DNA.

Analysis of hybridization values for markers from control DNA of *N. vitripennis* and *N. giraulti* showed largely nonoverlapping distributions (Figure 1)—DNA from *N. vitripennis* hybridized more strongly to the *N. vitripennis*-specific oligo 97.9% of the time, whereas DNA of *N. giraulti* hybridized more strongly to the *N. giraulti*-specific oligo 98.4% of the time. Furthermore, 19,681 of the 20,551 markers had median hybridization values for each species separated by at least 1 SD of each distribution, and 16,511 markers were separated by at least 2 SD. These largely nonoverlapping distributions of *N. vitripennis* and *N. giraulti* control DNA predict that we can call genotypes of unknown samples for most markers with high confidence.

### A new genetic map for Nasonia

**Figure 2:**
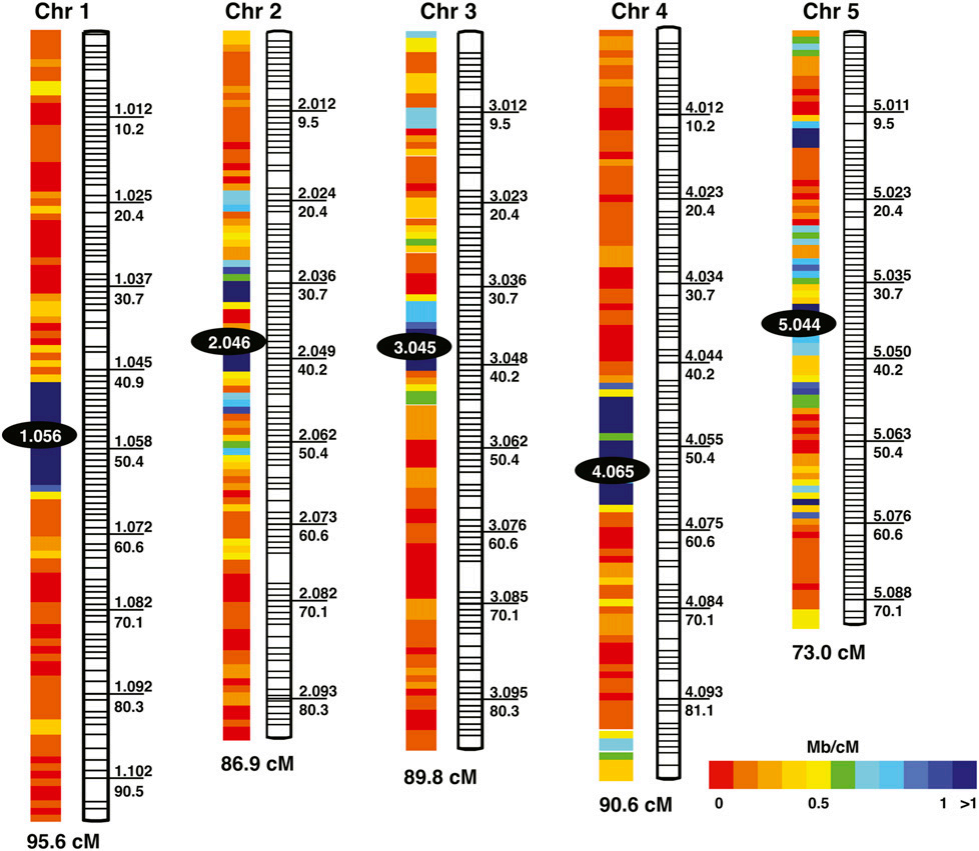
Genetic map of Nasonia. Marker cluster IDs are listed above the line to the right of each cluster, and corresponding map distances (in cM) are listed below the line. Recombination rates for each cluster are given in Table S1.

The genetic map constructed here contains 510 marker clusters mapped to five linkage groups (Figure 2), representing the five previously identified chromosomes of Nasonia (Rutten *et al.* 2004). Cluster- and marker-level details of the map are shown in Table S1 and Table S2, respectively. With respect to the Niehuis *et al.* (2010) map, the current map presented here expands the number of mapped scaffolds, increases the percentage of assembled genome that is mapped, and decreases average distance between clusters (see Table 3 for comparisons between the new and previous map). The 510 marker clusters in the new map almost doubles the 264 marker clusters of the Niehuis *et al.* (2010) genetic map. Despite the increase in mapped scaffolds and marker clusters, the recombination size of the new map totals 435.9 cM, similar to the 446.9 cM of the Niehuis *et al.* (2010) map. This finding suggests that we are converging on the recombination map size for Nasonia based on the hybrid mapping population.

**Table 3:** Comparison of new genetic map (2012) with the previous map (Niehuis *et al.* 2010)

			Chromosome									
	Total		1		2		3		4		5	
Map version	2010	2012	2010	2012	2010	2012	2010	2012	2010	2012	2010	2012
Genetic length, cM	446.9	435.9	94.5	95.6	89.8	86.9	98.9	89.8	87.6	90.6	76.1	73
No. observed recombinations	499	597	106	131	100	119	110	123	99	124	84	100
Marker clusters	264	510	61	108	58	102	53	104	41	104	51	92
Avg distance between marker clusters, cM	1.7	0.9	1.6	0.9	1.6	0.9	1.9	0.9	2.2	0.9	1.5	0.8
No. markers	1255	15546	372	4259	207	3435	188	3084	224	1930	264	2838
Mapped scaffolds	265	476	91	154	48	105	46	91	61	97	31	53
Mapped scaffolds (oriented)	42	53	11	15	10	10	9	9	5	11	7	8
Physical length, Mb	187.6	217.7	48.3	56.5	39.5	47.3	34	38	35.8	42.3	30	33.6
Associated markers	0	3702	0	657	0	478	0	411	0	1818	0	338
Associated additional scaffolds	0	253	0	63	0	39	0	51	0	82	0	23
Associated physical length, Mb	0	15.9	0	4.0	0	2.6	0	2.4	0	5.4	0	1.5
Total scaffolds	265	729	91	217	48	144	46	142	61	179	31	76
Total physical length, Mb	187.6	232.4	48.3	60.5	39.5	49.9	34	40.4	35.8	47.7	30	35.1

Results for the complete map are shown, as well as results for individual chromosomes. Key improvements in the new map include a dramatically increased marker density and the placement of a large number of small scaffolds.

Markers used to construct the genetic map represent 476 scaffolds (211 more than in the Niehuis *et al.* map), covering 74% of the assembled genome. Of these 476 scaffolds, 53 are oriented, while the others are placed within a cluster but not oriented because of absence of a recombination event within the scaffold that would allow orientation. We found evidence for 34 scaffolds being mis-assembled in the Nasonia genome assembly version 1.0 (Werren *et al.* 2010), and due to the high density of markers on the microarray, we are able to resolve breakpoints in scaffold assemblies to an average size of 52 Kb (Table S3).

Apparent in the genetic map (Table S1) is that typically one cluster per chromosome contains a large number of scaffolds and that many of these scaffolds are relatively short. These clusters likely correspond to regions containing the centromere and flanking heterochromatic DNA. The many small scaffolds can result from intervening repetitive DNA (*e.g.*, transposons or tandem repeats), typical of low recombination regions. Such features fail to assemble well in automated genome assemblies. Based on the criterion of a large number of scaffolds mapping to a single cluster, the clusters corresponding to the centromeric regions (and number of scaffolds within each one) for the five chromosomes are 1.056 (116), 2.046 (91), 3.045 (81), 4.065 (64), and 5.044 (43).

Because the total genetic length of the map did not change substantially between the new and previous maps (435.9 cM and 446.9 cM, respectively), the genome-wide recombination rate estimate of 1.4–1.5 cM/Mb made previously (Niehuis *et al.* 2010) is still accurate. Local recombination rates (see Table S1) were negatively correlated with both the number of scaffolds per cluster (Spearman’s rank order coefficient, r = −0.37, *P* < 0.001) and the number of markers per cluster (Spearman’s rank order coefficient, r = −0.58, *P* < 0.001). This finding likely reflects the accumulation of repetitive DNA in regions of low recombination as noted previously. Recombination rates on chromosome arms remained similar between the new and the previous map, which is expected as little sequence was added to chromosome arms in the new map. However, local recombination rates of centromeric clusters decreased dramatically, ranging from a 1.5-fold decrease at the chromosome 3 centromere to a 2.4-fold decrease at the chromosome 5 centromere due to the mapping of many smaller previously unmapped scaffolds to the centromeric region. Overall, the local recombination rates at the centromeres on the new map ranged from 0.044 to 0.173 cM/Mb, or 8.1- to 34.1-fold lower than the average recombination rate. Given that most of the remaining sequence that has still not been placed in the new map is repetitive and likely concentrated in the centromeres, the true recombination rate for centromeric regions is probably even lower than these new estimates.

To increase accuracy of the inferred map, certain markers were excluded during map reconstruction (see *Materials and Methods*). However, we were able to map many of these markers *a posteriori* to clusters on the linkage map. Table S4 shows the results for markers on scaffolds that are not represented on the genetic map, whereas Table S5 shows the corresponding information for markers on scaffolds represented on the genetic map. Of the 4135 unmapped markers, the genotype profile of 3702 matched that of at least one marker cluster within our mismatch threshold. This allows up to map an additional 253 scaffolds representing 4% of the assembled genome, bringing the complete map coverage to 729 scaffolds covering 79% of the assembled genome. Many of the previously unplaced scaffolds added here are short (median length 20.2 kb) and fall within low recombination regions.

### SILs for QTL mapping

**Figure 3:**
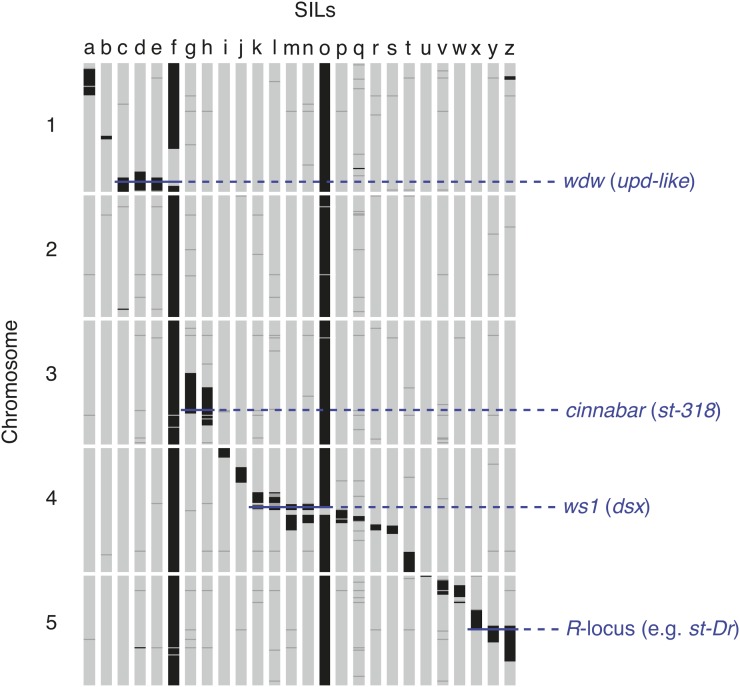
Genotype profiles of 26 SILs along the five chromosomes of Nasonia. Each cluster in the linkage map is depicted by a horizontal tick in black (*N. giraulti*), dark gray (ambiguous), or light gray (*N. vitripennis*). Clusters were assigned to a species when at least 60% of markers at that cluster were genotyped as that species; in cases in which neither species had at least 60% of markers, the cluster was labeled as ambiguous. Names and background information on the different SILs (a through z) are provided in Table 1.

To further develop resources for QTL mapping in Nasonia, we genotyped 26 SILs (summaries of the regions covered by SILs are described in Table 1 and depicted graphically in Figure 3). In total, the SILs cover 101 Mb (46%) of the mapped genome sequences. These SILs have proven useful in mapping QTL involved in species differences, and for mapping visible mutants generated in *N. vitripennis* by introgression of the wild-type *N. giraulti* allele into *N. vitripennis*. Examples of mapped QTL and visible mutants are shown in Figure 3. These SILs have been used as starting points for cloning the *widerwing* locus [*wdw*, which affects male wing size differences between *N. vitripennis* and *N. giraulti* and has been shown to be the growth regulator *unpaired-like* (Loehlin and Werren 2012)], cloning the male wing locus *wing-size-1* [*ws1*, shown to be the sex determining gene *doublesex* (Loehlin *et al.* 2010b)], in implicating *cinnabar* as the eye color mutant *st-318* (see the section *Bulk segregant analysis of a red eye mutant*), and in mapping the *R*-locus [(Saul 1990) a major nonrecombining locus containing genes affecting eye color and other phenotypes], to a region flanking the centromere on chromosome 5. These SILs and their extensive genotypic characterization can be used as an important resource for mapping additional QTL and identifying the underlying genes.

Some of these SILs are particularly helpful in resolving scaffold positions in regions of low recombination. Although recombination events in these regions are rare in the F_2_ mapping population, some SILs have been specifically generated in centromeric and flanking regions (*e.g.*, Desjardins *et al.* 2010; Loehlin *et al.* 2010b). Data from the SILs were used to more finely map scaffolds that had previously been assigned to a single cluster due to absence of F_2_ hybrids that are recombinant for these scaffolds (see *Materials and Methods*). From this analysis, we identified 12 additional clusters and were able to refine positions of 82 scaffolds between these clusters, thereby increasing linkage map resolution and accuracy. In three instances, we were also able to orient scaffolds because they crossed the recombination junction of the newly created clusters. Because recombination distances are estimated from the frequency of recombinant F_2_ hybrids, these clusters show no recombination between them in the linkage map.

### Bulk segregant analysis of a red eye mutant

Bulk segregant analysis is a method in which individuals in a population with segregating phenotypes are sorted into pools and then genotyped for associated molecular markers. To test the accuracy of this type of analysis with the microarray, we used a hybrid where wild-type *N. giraulti* were backcrossed in a *N. vitripennis* background with linked mutations *st-318* and *mm* (which create red and bulging eyes, respectively). Heterozygous females were then set onto hosts as virgins, and haploid male progeny recombinant between the two mutations were sorted into two sets: individuals with the wild-type *N. giraulti* st-318 phenotype and mutant *N. vitripennis* mm phenotype (*st-318^+g^*,*mm*) and individuals with the mutant *N. vitripennis* st-318 phenotype and wild-type *N. giraulti* mm phenotype (*st-318*,*mm^+g^*).

Each set was genotyped by both PCR and the microarray, and the results of are shown in Table 4. For all PCR markers, the proportion of each species allele in the bulk sample as estimated with the array is roughly equivalent to the proportion of each allele as determined by individual PCR. It should be noted that for these two measures to be theoretically equivalent, we assume that the DNA content of individuals in the bulk sample is independent of their genotype. As we are performing the analysis on adults, we also assume that survival to adulthood in these hybrid males is independent of their genotype at the loci under investigation. Therefore, this method should be used cautiously when the segregating phenotype causes large changes in body size or is associated with differential mortality. Pools should also be of either all male or all female samples to avoid differences due to ploidy levels (haploid males *vs.* diploid females).

**Table 4:** Allele composition of bulk segregant pools based on individual PCR and pooled microarray hybridization

Population	Scaffold	Position	Map Marker Cluster	PCR-based Proportion *N. vitripennis*	Array-based Proportion *N. vitripennis*	Map Markers within 100 kb
*st-318*,*mm^+g^*	22	1,033,000	3.053	0.17	0.12	11
	22	2,810,000	3.065	0.54	0.51	0*^a^*
	17	1,078,000	3.071	0.82	0.71	9
	17	1,504,000	3.078	1.00	1.00	10
*st-318^+g^*,*mm*	22	1,033,000	3.053	0.88	0.70	11
	22	2,810,000	3.065	0.58	0.56	0*^a^*
	17	1,078,000	3.071	0.24	0.28	9
	17	1,504,000	3.078	1.00	1.00	10

A mutant *N. vitripennis* strain (*st-318*,*mm*) was crossed with a wild-type *N. giraulti* introgression of the region (*st-318^+g^*,*mm^+g^*) in a *N. vitripennis* genetic background. Recombinants between the loci were collected into two pools: *st-318^+g^*,*mm* and *st-318*,*mm^+g^*. All members of each pool were screened with PCR markers at the listed positions to determine the PCR-based proportion of individuals with each allele. Then, each pool was hybridized to the array and allele proportions were estimated for all map markers. Array-based allele proportion at each PCR marker was determined by averaging proportions for all map markers in a 100-kb window surrounding the PCR marker location. PCR, polymerase chain reaction.

a Because no map markers were within 100 kb, we used the 10 closest map markers centered on the position.

We next used the microarray-based allele frequency estimates of the *st-318^+g^*,*mm* recombinants to identify a candidate gene for the st-318 red eye phenotype. These recombinants are all wild-type for *st-318* and therefore the pool of these individuals should have a near zero percentage of the (mutant) *N. vitripennis* allele directly around the *st-318* locus. However, because the wild-type *N. giraulti st-318* allele was backcrossed repeatedly into a *N. vitripennis* genetic background, the percentage of the *N. vitripennis* allele in the pool should rise sharply to near 100% at the edge of the introgressed region between the *st-318* locus and the end of the chromosome. Additionally, because each individual in the pool has a (mutant) *N. vitripennis mm* allele, the percentage of the *N. vitripennis* allele should rise gradually toward 100% along the chromosome approaching the *mm* locus.

**Figure 4:**
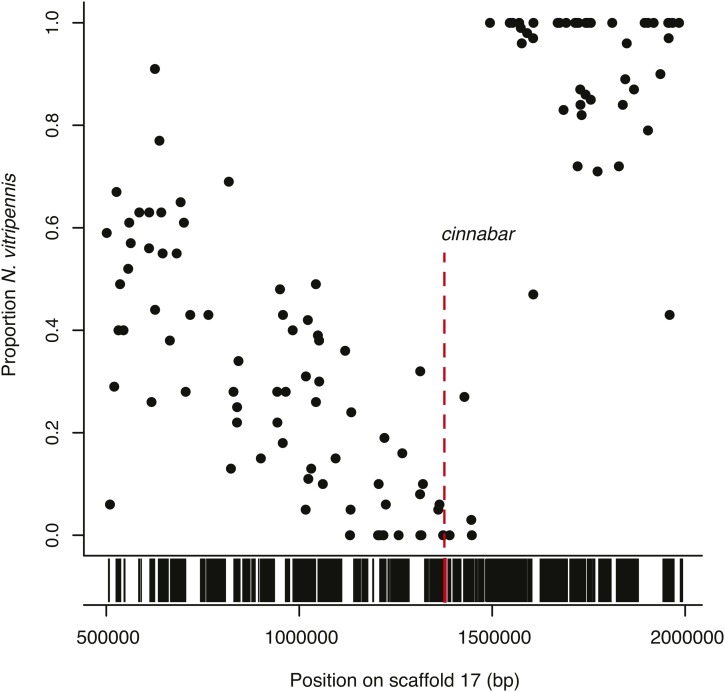
Bulk segregant analysis suggesting that the st-318 red-eye phenotype is caused by the gene *cinnabar*. A mutant *N. vitripennis* strain (*st-318*,*mm*) was crossed with a wild-type *N. giraulti* introgression of the region (*st-318^+g^*,*mm^+g^*) in a *N. vitripennis* genetic background. Twenty-four recombinants with the wild-type *N. giraulti* st-318 phenotype and mutant *N. vitripennis* mm phenotype (*st-318^+g^*,*mm*) were collected, pooled, and genotyped. Shown here is the proportion of sample with *N. vitripennis* genotype for markers across scaffold 17, 50−200 Kb in the *st-318^+g^*,*mm* pool. Because all recombinants show the wild-type *N. giraulti* st-318 phenotype, the proportion of individuals with *an N. vitripennis* genotype should approach zero at the site of *st-318* locus. Gene presence along the same region is shown in black bars below. *Cinnabar*, a gene known to cause a red-eye phenotype, is indicated in red and is located in the noted region.

Estimated proportions of *N. vitripennis* alleles in *st-318^+g^*,*mm* recombinants for markers on scaffold 17, which contains the region under study, are shown graphically in Figure 4. In the *st-318^+g^*,*mm* recombinants the proportion of the *N. vitripennis* alleles declines steadily along scaffold 17 until around 1.44 Mb, where the proportion approaches zero before sharply rising to 100% *N. vitripennis* at 1.49 Mb. As this sharp increase should be near the *st-318* locus, we examined this region and noted the gene *cinnabar* at 1.38 Mb. *Cinnabar* is an eye color gene which is already known to produce a red-eye phenotype after RNAi knockdown (Werren *et al.* 2009), and we therefore hypothesize that the st-318 phenotype is the result of a mutation in this gene. Here, hybridization of a single sample to the array has accurately estimated the true proportion of alleles belonging to each genotype along the region of interest, allowing a causal gene to be hypothesized. A similar approach was not employed with *mm* due to variable expressivity of the mutation and low recombination rate in the region.

### Extrapolation to other Nasonia species

**Figure 5:**
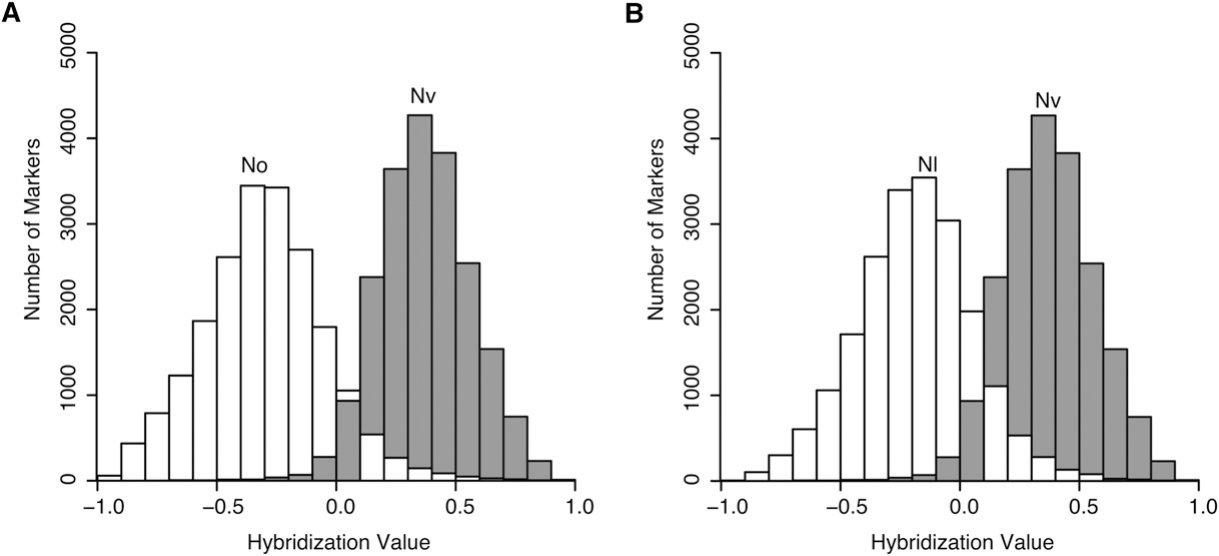
Frequency histograms of hybridization values when hybridized with (A) *N. oneida* (No; white) *vs. N. vitripennis* (Nv; gray), and (B) *N. longicornis* (Nl; white) *vs. N. vitripennis* (Nv; gray). The Y-axis shows the number of markers that produced the relevant hybridization values of the 20,551 total markers. Hybridization values > 0 indicate greater hybridization to the *N. vitripennis* oligo, while hybridization values < 0 indicate greater hybridization to the *N. giraulti* oligo. A large fraction of markers successfully discriminate between the two species pairs, as most markers produced greater hybridization to the correct oligo when hybridized with control DNA.

We tested the ability of the array to distinguish between other Nasonia species pairs. Similar to the original *N. vitripennis*−*N. giraulti* comparisons, hybridization values for *N. vitripennis*−*N. oneida* and *N. vitripennis*−*N. longicornis* comparisons showed largely non-overlapping distributions with *N. vitripennis* (Figure 5). For the *N. vitripennis*−*N. oneida* and *N. vitripennis*−*N. longicornis* comparisons, 91.4% and 85.5% of markers, respectively, had median hybridization values separated by at least 1 SD. This result correlates with the phylogenetic relationships of these species, *i.e.*, *N. giraulti* and *N. oneida* are more closely related to each other than to *N. longicornis* (Raychoudhury *et al.* 2010). Furthermore, it suggests that although there is some loss of useful markers relative to the original *N. vitripennis*−*N. giraulti* comparison, the array can still effectively genotype a large number of markers in *N. vitripennis*−*N. oneida* and *N. vitripennis*−*N. longicornis* comparisons. To further evaluate the use of the array for genotyping *N. vitripennis*−*N. longicornis* hybrids, we calculated precision to be 98.4% and recall to be 91.2% for a SIL from this species pair (SIL17_ws1_lV), demonstrating that much of the array can effectively distinguish between *N. vitripennis* and *N. longicornis* DNA. Finally, the array was tested for its ability to distinguish genotypes of hybrids between the two more closely related species *N. giraulti* and *N. longicornis*. In this case, only 38.2% of markers had median hybridization values separated by at least at least one standard deviation. Although it may be possible to genotype large stretches of DNA in *N. giraulti*−*N. longicornis* hybrids, the array likely is of limited use for genotyping hybrids of Nasonia species pairs that do not involve *N. vitripennis*.

### Conclusions

A CGH array containing more than 20,000 markers representing SNP-cluster and indel polymorphisms between *N. vitripennis* and *N. giraulti* has been successfully used to create a high-resolution linkage map of Nasonia, covering 79% of the assembled genome of *N. vitripennis*. Errors in the original *N. vitripennis* assembly have been identified through mapping, and this information can be used to improve the next assembly. The CGH array also provides an inexpensive means of mapping QTL involved in species differences and visible mutants onto the linkage map. The array can be used for bulk-segregant analysis, as was demonstrated by identifying the gene *cinnabar* as a potential cause of the red eye mutant *st-318*. Hybrids of *N. vitripennis−N. oneida* and *N. vitripennis−N. longicornis* species pairs also can be genotyped using the microarray due to shared fixed marker differences between *N. longicornis*, *N. oneida*, and *N. giraulti*. The genetic map and 26 SILs presented here will further promote Nasonia as a model for studying the genetics of complex traits.

The microarray design, including oligo sequences and SNP information is available by request from the authors. Perl scripts for processing data are available as File S2, and additional scripts for data analysis are available by request from the authors. Segmental introgression lines reported here can be obtained by contacting J. H. Werren (werr@mail.rochester.edu).

## Supplementary Material

Supporting Information
