# Supplementary material for: Fine-Scale Mapping of the Nasonia Genome to Chromosomes Using a High-Density Genotyping Microarray
Source: G3 (Bethesda). 2013 Feb 1;3(2):205–15. doi: 10.1534/g3.112.004739 (PMC3564981; doi:10.1534/g3.112.004739)
Supplement: Supporting Information [file supp_3_2_205__index.html]

Supporting Information 

# Fine-Scale Mapping of the Nasonia Genome to Chromosomes Using a High-Density Genotyping Microarray

## Supporting Information for Desjardins *et al.*, 2013

**Files in this Data Supplement:**

- Supporting Information - Figure S1, Files S1-S2, and Tables S1-S6 (PDF, 166 KB)
- Figure S1 - Effect of varying genotyping and oligo-pair selection parameters on precision and recall (PDF, 103 KB)
- File S1 - Genotype Calling and Parameter Optimization Text (PDF, 93 KB)
- File S2 - Processing data from the NimbleGen CGH *Nasonia* Microarray array using nasoniaGenotyper\_v0.94 (.zip, 2 MB)
- Table S1 - Cluster-level details of the genetic map (.xlsx, 59 KB)
- Table S2 - Marker-level details of the genetic map (.xlsx, 533 KB)
- Table S3 - Mis-assemblies identified by new map (.xlsx, 45 KB)
- Table S4 - Position of markers from scaffolds not on the genetic map (.xlsx, 18 KB)
- Table S5 - Position of markers from scaffolds on the genetic map, but with ambiguous genotypes (.xlsx, 120 KB)
- Table S6 - Effect of varying genotyping parameters on precision and recall (.xlsx, 99 KB)
