# Supplementary material for: Fine-Scale Mapping of the Nasonia Genome to Chromosomes Using a High-Density Genotyping Microarray
Source: G3 (Bethesda). 2013 Feb 1;3(2):205–15. doi: 10.1534/g3.112.004739 (PMC3564981; doi:10.1534/g3.112.004739)
Supplement: Supporting Information [file supp_3.2.205_004739SI.pdf]

G3 · Genes | Genomes | Genetics

Fine Scale Mapping of the Nasonia Genome to Chromosomes Using a High-Density Genotyping Microarray

Christopher A. Desjardins*[,†,1], Jürgen Gadau[‡], Jacqueline A. Lopez[§,2], Oliver Niehuis[‡,**], Amanda R. Avery*, David W. Loehlin*[,3], Stephen Richards[††], John K. Colbourne[§,4], John H. Werren*

*Department of Biology, University of Rochester, Rochester, NY 14627
[†]Broad Institute, Cambridge, MA 02141
[‡]School of Life Sciences, Arizona State University, Tempe, AZ 85287
[§]The Center for Genomics and Bioinformatics, Indiana University, Bloomington, IN 47405
**Center for Molecular Biodiversity Research, Zoological Research Museum Alexander Koenig, 53113 Bonn, Germany
[††]Human Genome Sequencing Center, Baylor College of Medicine, Houston, TX 77030

[1]Corresponding author. E-mail: cdesjard@broadinstitute.org
[2]Present address: Department of Biological Sciences, University of Notre Dame
Notre Dame, IN 46556
[3]Present address: Howard Hughes Medical Institute and Laboratory of Molecular Biology, University of Wisconsin, Madison, WI 53706
[4]Present address: School of Biosciences, The University of Birmingham
Birmingham, B15 2TT, United Kingdom

DOI: 10.1534/g3.112.004739

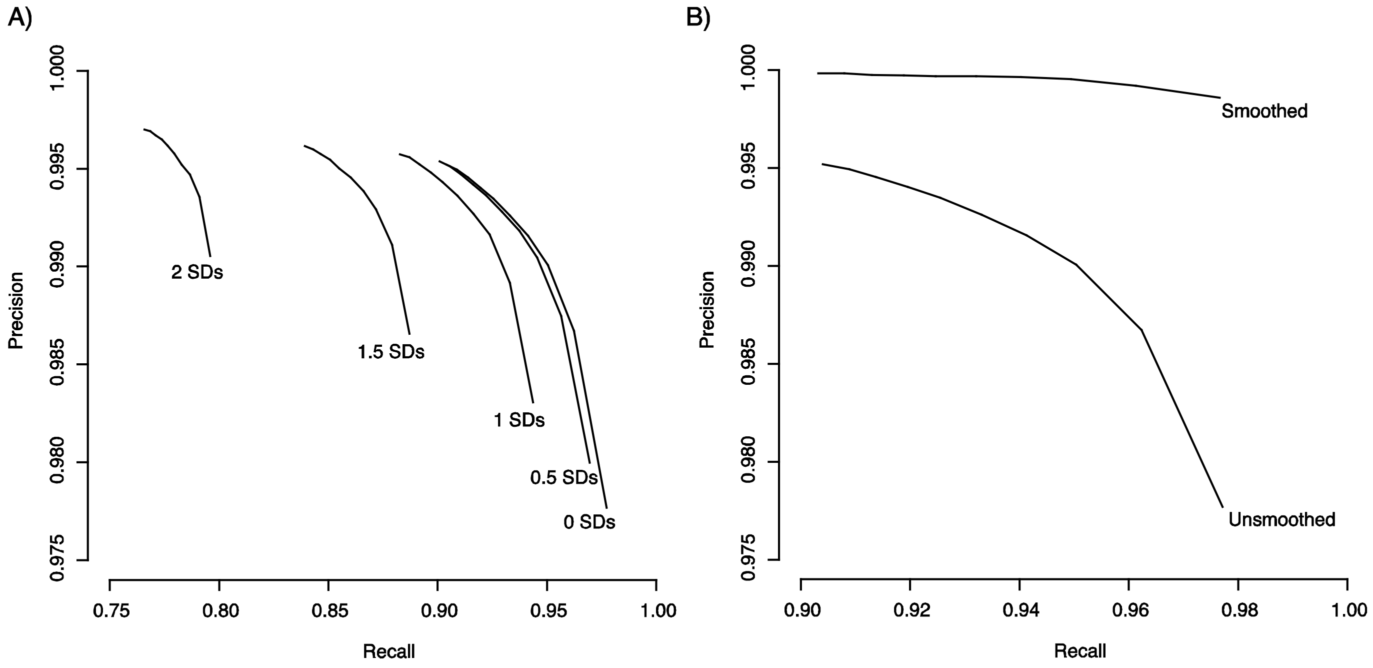

Figure S1 Effect of varying genotyping and oligo-pair selection parameters on precision and recall. Calculations were made based on 30 independently genotyped individuals, with the minimum p-value genotyping parameter set to 1x10-5. (A) Effect of varying the genotyping parameter minimum probability ratio (mpr, along lines) and oligo selection parameter minimum separation (SDs, between lines). Increasing both parameter results in increases in precision, but increasing minimum separation results in a greater loss in recall than increasing minimum probability ratio. (B) Effect of using the smoothing algorithm, while varying minimum probability ratio and with minimum separation set to 0 SDs. Smoothing dramatically increases precision at even the lowest parameter settings, yet retains a high degree of recall.

File S1

Genotype Calling and Parameter Optimization Text

Genotype Calling

We developed three parameters to assist in genotype calling. The first parameter, called *minimum separation*, is used to exclude poorly performing markers prior to genotyping. These poorly performing markers are identified by their inability to properly distinguish between the two species in control experiments. The parameter is defined as the minimum separation, in standard deviations (SDs), of the medians of the pure species hybridization value distributions required for a marker to be considered reliable. For example, a minimum separation of 1 SD means the median hybridization values for *N. vitripennis* (V) and *N. giraulti* (G) must be separated by 1 V SD + 1 G SD.

The second and third parameters do not exclude markers prior to genotyping, but rather determine how strong the evidence is required to be in order to call a specific genotype (V or G) over an ambiguous genotype (U). The second parameter, *minimum probability ratio*, is the minimum ratio of the cumulative probability that the sample is the more likely genotype to the cumulative probability that the sample is the less likely genotype ((max(p(V),p(G))/(min(p(V),p(G))) required for the sample to be called a non-ambiguous genotype. For example, a minimum probability ratio of two means that the more likely genotype must be at least twice as likely as the less likely genotype in order for a non-ambiguous genotype call to be made. This parameter is intended to prevent non-ambiguous calls when genotype evidence is amibiguous.

The third parameter, *minimum p-value*, is the minimum p-value (cumulative probability) of the more probable genotype (max(p(V),p(G)) required for a sample to be called the more probable genotype rather than ambiguous. For example, a minimum p-value of 0.05 means that if a sample has cumulative probabilities below 0.05 for both the V or G genotype, it will be called ambiguous. This parameter is intended to prevent non-ambiguous calls when data are unlikely to belong to either genotype.

Parameter Optimization

It was then important to select the optimal values of each parameter to maximize precision (the probability that a called genotype is correct) and recall (the probability that a non-ambiguous genotype is called for a given marker, also referred to as sensitivity). The relative weight of precision and recall can be balanced in F-scores, where the subscript of the F-score represents the relative weight of precision versus recall. For F_1, both precision and recall are weighted

equally, while for $F_{0.5}$, precision is weighted relatively greater than recall. Optimization of F-scores determines the values of parameters that optimize precision and recall simultaneously according to their relative weights.

In order to calculate F-scores for different parameter values, we used an independently genotyped population of 30 hybrid individuals (Niehuis *et al.* 2010). For each chromosome, we identified a 2-Mb stretch contained within a single large scaffold. Then for each individual, if all markers within a given 2-Mb stretch plus the neighboring marker on either side were called the same genotype by Niehuis *et al.* (2010), that region was included in this analysis. In total, these regions contained 26,578 testable markers. We then tested genotyping accuracy by varying the three parameters and genotyping the control markers. The parameter ranges tested were 0–2 SDs for minimum separation, 1:1–10:1 for the minimum probability ratio, and 10^{-7}–10^{-3}, for the minimum p-value

Results of these optimizations are shown in Table S6, and the effects of varying the two parameters with the greatest influence on precision, the minimum separation, and minimum probability ratio are shown in Figure S1A. When precision and recall are weighted equally, F_1 is maximized with the following parameter settings: minimum separation = 0 SDs, minimum probability ratio = 1:1, and minimum p-value = 10^{-7}. At these settings, precision is 97.8% and recall is 97.7%. This suggests that the most permissive parameter settings should be used when calling a maximum number of genotypes is just as important as having those calls be correct.

However, it is often the case that precision is more important than recall. When precision is given greater weight, e.g., $F_{0.25}$, the optimal parameters change to minimum separation = 0, minimum probability ratio = 9:1, and minimum p-value = 10^{-5}. At these settings, precision is 99.5% and recall is 90.8%. This analysis shows that increasing the minimum probability ratio increases precision at the smallest cost to recall of the three parameters. Increasing the minimum separation or minimum p-value does also increase precision, but with a large corresponding loss to recall. The lack of effect of increasing minimum separation of the two species distributions in the control data suggests that to some extent, it is not effective to pre-filter a large number of markers based on performance with control data. Rather, a greater overall precision can be achieved by attempting to genotype a large fraction of the markers with more stringent criteria.

In an attempt to increase precision beyond 99.5%, we developed a smoothing algorithm based on genotypes of neighboring markers (see Methods). To determine the effectiveness of the smoothing, we replicated the optimization conducted for the unsmoothed data and compared the two (Table S6, Figure S1B). Smoothing dramatically affects all F-scores, which are maximized at minimum separation = 0 SDs, minimum probability ratio = 1:1, and minimum p-value ranging from 10^{-7} to 10^{-5}, depending on the F number. In all cases, smoothed data have

higher F-scores than unsmoothed data. Under these parameters, precision is 99.9% and recall is 97.7%. This indicates that the smoothing algorithm dramatically increases precision without the loss in recall seen when increasing the minimum probability ratio in the absence of smoothing. Therefore it is recommended that the smoothing algorithm is always used. It should be noted however, that smoothing does not affect scaffolds with only one marker, so the F-scores for these scaffolds will be the same as for unsmoothed data. Therefore, optimal parameter setting depends on the user's relative interest in the accuracy of single marker scaffolds versus regions with higher overall marker density.

File S2

Processing data from the NimbleGen CGH *Nasonia* Microarray array using nasoniaGenotyper_v0.94

File S2 is available for download at http://www.g3journal.org/lookup/suppl/doi:10.1534/g3.112.004739/-/DC1/FileS2.zip.

Tables S1-S6

Available for download at http://www.g3journal.org/lookup/suppl/doi:10.1534/g3.112.004739/-/DC1

Table S1: Cluster-level details of the genetic map

Table S2: Marker-level details of the genetic map

Table S3: Mis-assemblies identified by new map

Table S4: Position of markers from scaffolds not on the genetic map

Table S5: Position of markers from scaffolds on the genetic map, but with ambiguous genotypes

Table S6: Effect of varying genotyping parameters on precision and recall
