# Supplementary material for: Fine-Scale Mapping of the Nasonia Genome to Chromosomes Using a High-Density Genotyping Microarray
Source: G3 (Bethesda). 2013 Feb 1;3(2):205–15. doi: 10.1534/g3.112.004739 (PMC3564981; doi:10.1534/g3.112.004739)
Supplement: Supporting Information [file supp_3.2.205_FigureS1.pdf]

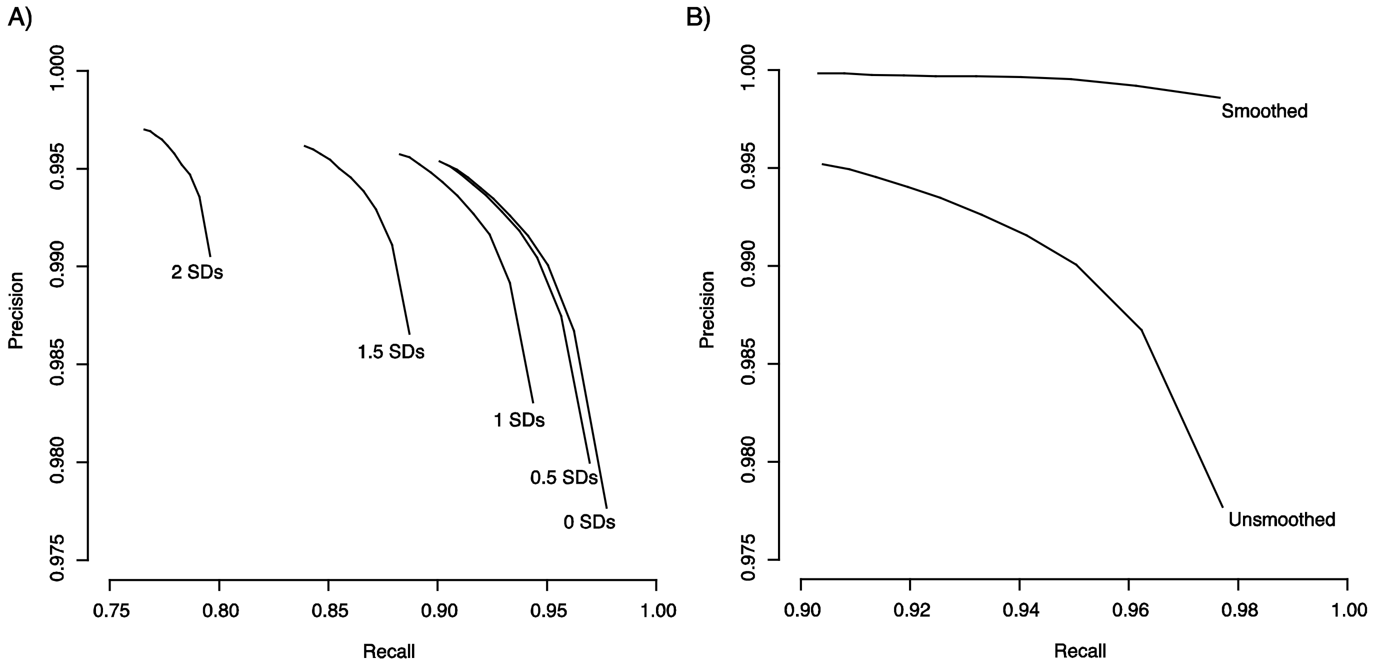

Figure S1 Effect of varying genotyping and oligo-pair selection parameters on precision and recall. Calculations were made based on 30 independently genotyped individuals, with the minimum p-value genotyping parameter set to 1x10-5. (A) Effect of varying the genotyping parameter minimum probability ratio (mpr, along lines) and oligo selection parameter minimum separation (SDs, between lines). Increasing both parameter results in increases in precision, but increasing minimum separation results in a greater loss in recall than increasing minimum probability ratio. (B) Effect of using the smoothing algorithm, while varying minimum probability ratio and with minimum separation set to 0 SDs. Smoothing dramatically increases precision at even the lowest parameter settings, yet retains a high degree of recall.
