# Supplementary material for: Fine-Scale Mapping of the Nasonia Genome to Chromosomes Using a High-Density Genotyping Microarray
Source: G3 (Bethesda). 2013 Feb 1;3(2):205–15. doi: 10.1534/g3.112.004739 (PMC3564981; doi:10.1534/g3.112.004739)
Supplement: Supporting Information [file supp_3.2.205_FileS2.zip › nasoniaGenotyper_v0.94/nasoniaGenotyper_v0.94.pdf]

Processing data from the NimbleGen CGH *Nasonia* Microarray array using nasoniaGenotyper_v0.94

Christopher Desjardins and Jacqueline Lopez
5/3/12

Introduction

nasoniaGenotyper_v0.94 is a beta version of PERL tools for processing data from the NimbleGen *Nasonia* genotyping array. If you have questions or comments please email Christopher Desjardins <cdesjard@broadinstitute.org>.

Installation

All the scripts for processing data are written in PERL, so you will need a PERL installation to run them. Additionally, you will need to install the PERL module Math::Cephes from CPAN. You may also need to install Statistics::Descriptive and FindBin, but they are likely to be included in your default PERL installation. The processing scripts described here come in a directory nasoniaGenotyper_v0.93 which includes this document, a library directory "arrayLib" and the following 3 control scripts:

**Control Scripts**
processNasoniaArray.pl
callNasoniaGenotypes.pl
callNasoniaProportions.pl

**arrayLib contents**
Desjardins_markers.txt
arrayPropIV.pl
array_controls_nvng_md_0sd_120811.info
array_controls_nvnl_mn_0sd_120811.info
array_controls_nvno_md_0sd_120811.info
genotypeCallerV.pl
genotypes2map.pl
indel_oligo.info
oligo2scaff.link
processRawArrayII.pl
smoothGenotypesIII.pl

Processing Raw Data

Raw data for the NimbeleGen CGH *Nasonia* Microarray is stored as a PAIR Report (.pair). A pair report is a tab-delimited text file which provides sequence, probe, and signal intensity information for each data channel. The report contains a single report header line (marked with a #) followed by a single column header line containing the field names identifying 10 columns of data (NimbleScan Software User's Guide, Roche NimbleGen, Version 2.6, March 2010, p. 114). The pair report is processed with the script processNasoniaArray.pl.

```
Usage:
 processNasoniaArray.pl file_of_filenames

Required Input:
  file_of_filenames = list of .pair files, one filename per line
```

Output from the processNasoniaArray.pl script is in the form of a DATA Report (.data). A data report is a tab delimited file with a single column header line containing the field names identifying 4 columns of data.

**Column**	**Description**
Oligo_Name	PROBE_ID within .pair file. The NimbleGen probe identifier of the *vitripennis* allele of a locus.
(V-G)/(V+G)	Differential hybridization, calculated as (Raw_V - Raw_G)/(Raw_V + Raw_G)
Raw_V	PM within .pair file. Raw intensity of hybridization to *vitripennis* allele for that locus.
Raw_G	PM within .pair file. Raw intensity of hybridization to *giraulti* allele for that locus.

Calling Genotypes: Theory

In order to call a genotype for a given locus of a test individual, we calculate the cumulative probability that the locus belongs to each genotype based on control data from pure species, assuming the control data is normally distributed.

Because some level of miscalling is inevitable at the level of individual markers, we have added a second step where we “smooth” genotype calls based on the calls of their neighbors. The rules for smoothing are simple: If a scaffold has only 1 locus, no correction can be made. If a scaffold has 2 loci, and those loci are called opposite genotypes, both are converted to ambiguous. If the scaffold has 3 or more loci, for each locus, if 1 of the two closest loci is the opposite genotype and the other is either the opposite genotype or ambiguous, that locus is converted to ambiguous.

Calling Genotypes: How To

To call genotypes on DNA from an individual *Nasonia*, use the script callNasoniaGenotypes.pl. Note that this script uses the Perl module Math::Cephes which must be installed for the script to work properly.

```
Usage:
 callNasoniaGenotypes.pl file_of_datafilenames oligo_control_file
confidence min_prob

Required Input:
 file_of_datafilenames = list of .data files from
processNasoniaArray.pl, one filename per line
 oligo_control_file = see table below, choose only one
 confidence = Numeric value, >=1, that defines the minimum ratio
of (max(prob(V),prob(G))/(min(prob(V),prob(G)))
 min_prob = Numeric value, >0, that defines the minimum
probability needed to call a genotype
```

Choosing an oligo_control_file: This file gives information on how well *vitripennis* and *giraulti* (or an alternative species) controls hybridize to the oligos. Select the file appropriate to your comparison.

Choosing confidence: Confidence is defined as the minimum ratio of (max(prob(V),prob(G))/(min(prob(V),prob(G))) in order for a genotype to be called. In other words, when confidence is set to 2, one genotype must be twice as probable as the other for the former genotype to be called. Confidence = 1 is the default value. How you should set your confidence depends largely on how important very small scaffolds (those with only 1 marker) are to you. If they are not important, you can get very high accuracy on a large proportion of loci using the default settings and smoothed genotype calls. However, single locus scaffolds cannot take advantage of the smoothing process, so if these scaffolds are the focus of your investigation, confidence = 9 should be used to get improved accuracy on those scaffold calls, although there will be many more ambiguous genotype calls overall.

Choosing min_prob: Min_prob is the minimum probability needed to call a genotype. min_prob = 0.00001 is the default value.

The script callNasoniaGenotypes.pl generates two forms of output: 1) a CALLS Report (.calls), which contains detailed genotyping information for all loci on the array, and 2) a MAPPED report, which calls genotypes for each locus on the genome map.

A .calls report is a tab delimited file with a single column header line containing the field names identifying 9 columns of data.

**Column**	**Description**
Oligo_Name	PROBE_ID within .pair file. The NimbleGen probe identifier of the *vitripennis* allele of a locus.
Scaffold_Name	The scaffold on which the probe is located.
Oligo_Start	An integer that indicates the start position of the PROBE_SEQUENCE in the target sequence or region of interest, starting at the 5' end.
Oligo_Stop	An integer that indicates the stop position of the PROBE_SEQUENCE in the target sequence or region of interest, starting at the 5' end.
Probability_V	Probability of the sample belonging to genotype V (*vitripennis*) based on control data from pure species
Probability_G	Probability of the sample belonging to genotype G (*giraulti* or alternate species) based on control data from pure species
Confidence_Ratio	max(Probability_V,Probability_G)/min(Probability_V,Probability_G)
Orig_Call	genotype call of V (*vitripennis*), G (*giraulti* or alternate species), or U (ambiguous) based on the the Probability_V and Probability_G
Smoothed_Call	genotype call based on Orig_Call and calls of neighbors of the locus

A .mapped report is a tab delimited file with a single column header line containing the field names identifying 5 columns of data.

**Column**	**Description**
Marker_Cluster	Numerical Marker ID on the genome map.
Cluster_cM	cM position along the chromosome.
Marker_Name	Marker name giving the location of the marker on the genome sequence.
Marker_Number	Sequential numbering of markers on the genome map.
Genotype	genotype call of V (*vitripennis*), G (*giraulti* or alternate species), or U (ambiguous) taken from Smoothed_Call in .calls report.

Estimating Proportions From Bulk Samples: Theory
When hybridizing a bulk sample (a single sample containing DNA from many individuals) to the genotyping array, we want to estimate the relative proportions of the 2 genotypes rather than call one genotype or the other. In order to do this, we assume that the proportion of each allele in the sample is uniformally distributed between the medians of each set of pure species control data.

Estimating Proportions From Bulk Samples: How To
To estimate allele proportions in a sample proportion use the script callNasoniaProportions.pl.

```
Usage:
 callNasoniaProportions.pl file_of_datafilenames
oligo_control_file prop

Required Input:
 file_of_datafilenames = list of .data files from
processNasoniaArray.pl, one filename per line
 oligo_control_file = see table above, choose only one
 prop = "prop", method to determine proportions
```

The script callNasoniaProportions.pl generates two forms of output: 1) a PROP Report (.prop), which contains detailed proportion information for all loci on the array, and 2) a MAPPED report, which shows proportions for each locus on the genome map.

A .prop report is a tab delimited file with a single column header line containing the field names identifying 5 columns of data.

**Column**	**Description**
Oligo_Name	PROBE_ID within .pair file. The NimbleGen probe identifier of the vitripennis allele of a locus.
Scaffold_Name	The scaffold on which the probe is located.
Oligo_Start	An integer that indicates the start position of the PROBE_SEQUENCE in the target sequence or region of interest, starting at the 5' end.
Oligo_Stop	An integer that indicates the stop position of the PROBE_SEQUENCE in the target sequence or region of interest, starting at the 5' end.
Proportion_V	Estimate of the proportion of the *vitripennis* genotype in the sample.

A .mapped report is a tab delimited file with a single column header line containing the field names identifying 5 columns of data.

**Column**	**Description**
Marker_Cluster	Numerical Marker ID on the genome map.
Cluster_cM	cM position along the chromosome.
Marker_Name	Marker name giving the location of the marker on the genome sequence.

Marker_Number	Sequential numbering of markers on the genome map.
Proportion_V	Estimate of the proportion of the *vitripennis* genotype in the sample.
